# The Potential Role of Phenolic Acids from *Salvia miltiorrhiza* and *Cynara scolymus* and Their Derivatives as JAK Inhibitors: An In Silico Study

**DOI:** 10.3390/ijms23074033

**Published:** 2022-04-05

**Authors:** Hui-Jun Liao, Jason T. C. Tzen

**Affiliations:** Graduate Institute of Biotechnology, National Chung Hsing University, Taichung 402202, Taiwan; tctzen@dragon.nchu.edu.tw

**Keywords:** Janus kinase, JAK, Cynarin, Salvianolic acid, *Salvia miltiorrhiza*, Danshen, *Cynara scolymus*, artichoke, benzofuran, AutoDock Vina

## Abstract

JAK inhibition is a new strategy for treating autoimmune and inflammatory diseases. Previous studies have shown the immunoregulatory and anti-inflammatory effects of *Salvia miltiorrhiza* and *Cynara scolymus* and suggest that the bioactivity of their phenolic acids involves the JAK-STAT pathway, but it is unclear whether these effects occur through JAK inhibition. The JAK binding affinities obtained by docking Rosmarinic acid (RosA), Salvianolic acid A (SalA), Salvianolic acid C (SalC), Lithospermic acid, Salvianolic acid B and Cynarin (CY) to JAK (PDB: 6DBN) with AutoDock Vina are −8.8, −9.8, −10.7, −10.0, −10.3 and −9.7 kcal/mol, respectively. Their predicted configurations enable hydrogen bonding with the hinge region and N- and C-terminal lobes of the JAK kinase domain. The benzofuran core of SalC, the compound with the greatest binding affinity, sits near Leu959, such as Tofacitinib’s pyrrolopyrimidine. A SalC derivative with a binding affinity of −12.2 kcal/mol was designed while maintaining this relationship. The docking results show follow-up studies of these phenolic acids as JAK inhibitors may be indicated. Furthermore, derivatives of SalC, RosA, CY and SalA can yield better binding affinity or bioavailability scores, indicating that their structures may be suitable as scaffolds for the design of new JAK inhibitors.

## 1. Introduction

### 1.1. Natural Substances Involved in JAK-STAT Pathway

Janus kinases (JAKs) are a family of intracellular tyrosine kinases that mediate the down transduction of Type I/II cytokine signals via the JAK-STAT (signal transducer and activator of transcription) pathway. A variety of autoimmune diseases, inflammatory skin diseases and cancers were found to be involved in the overexpression of Type I/II cytokines and the activation of the JAK/STAT pathway. JAKs’ inhibition is intended to limit this transmission to abate related diseases [1,2,3]. Tofacitinib is currently the most important oral JAK inhibitor in clinical medicine. Tofacitinib was approved in 2012 by the FDA for use to treat adults with moderate to severe rheumatoid arthritis who have had an inadequate response to, or who are intolerant of, methotrexate [4]. Tofacitinib is now also available for the treatment of psoriasis, psoriatic arthritis and ulcerative colitis [5,6].

Many natural substances, such as resveratrol, curcumin, caffeic acid and silibinin, have been reported to be involved in the JAK-STAT pathway in a manner beneficial to cancer prevention and treatment [7]. Vadapalli, J., et al. found that the polyphenol “Rottlerin” isolated from *Mallotus philippensis* is a potent JAK2 kinase inhibitor with a good binding affinity (−9.3 kcal/mol) [8]. Similar to polyphenols, phenolic acids are important healthy natural substances. *Salvia miltiorrhiza* (*S. miltiorrhiza* or Danshen) and *Cynara scolymus* (artichoke) are two natural products that are rich in phenolic acids and are widely cultivated in the field [9,10,11,12]. *S. miltiorrhiza*, also known as Chinese sage, has seen medicinal use for over 2000 years, and is best known for its cardiovascular health benefits [13]. The water-soluble phenolic acids of *S. miltiorrhiza* include Danshensu, Rosmarinic acid, Lithospermic acid, Salvianolic acid A-K, etc. Salvianolic acid B is the most abundant component in Danshen tea. Recent studies have found that *S. miltiorrhiza* has a modulating effect on immune diseases of interest to JAK inhibitors, such as improving rheumatoid arthritis [14]. Further studies have shown that Salvianolic acids can reduce inflammation and effectively downregulate cytokines [15,16,17,18]. Salvianolic acid B can suppress JAK-STAT1 activation by inhibiting the IFN-γ-induced phosphorylation of JAK2 (Tyr 1007/1008) and STAT1 [19]. *Cynara scolymus* (artichoke) is an indispensable vegetable on the Italian table and also has a place in traditional European medicine. Modern research has shown that artichoke extract has health effects such as protecting the liver, preventing arteriosclerosis and regulating immunity [20,21,22]. A series of caffeic acid derivatives are important constituents of artichokes, the most prominent of which are Cynarin (Cynarine or 1,3-Dicaffeoylquinic acid) and Chlorogenic acid. According to the study by Pandino, G. et al., Cynarin content in the dry matter of artichoke reaches 6500~8000 mg/kg [23]. Cynarin has been shown to suppress cancer cell proliferation through the IL6/JAK2/PI3K pathway [24]. The bioactivities of *Salvia miltiorrhiza* and *Cynara scolymus* involve the JAK/STAT pathway, but it is unclear whether their phenolic acids can directly inhibit JAK. Rosmarinic acid (RosA), Lithospermic acid (LSA) and Salvianolic acids A, B and C (SalA~C) and Cynarin (CY) are six widely studied antioxidant compounds found in these plants. The binding affinities and lowest-energy configurations of these phenolic acids to JAK can be obtained by molecular modeling to assess their potential for JAK inhibition.

### 1.2. JAK Family and JAK Inhibitors

**Figure 1:**
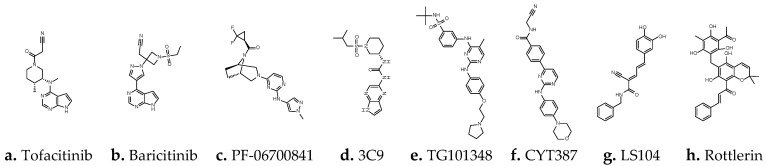
A series of JAK inhibitors.

**Figure 2:**
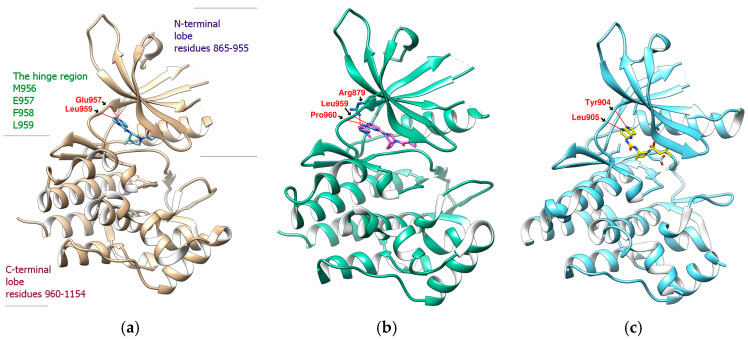
JAK inhibitor (ligand) and JAK complex. (**a**) Tofacitinib/PDB:3EYG(JAK1/2); (**b**) PF–06700841/PDB:6DBN(JAK1); (**c**) 3C9/PDB:4QT1(JAK3). Hydrogen bonds were shown as red lines.

The JAK family has four members: JAK1, JAK2, JAK3 and TYK2. JAK1/2 (PDB:3EYG), JAK1 (PDB:6DBN), and JAK3 (PDB:4QT1) contain representative JAK inhibitors (Figure 1 and Figure 2) [25,26,27]. PDB: 3EYG is the crystal structure of Tofacitinib complexed to the JAK1/2 protein tyrosine kinase domains. Tofacitinib takes pyrrolopyrimidine as the pharmacodynamic subunit to establish hydrogen bonds with Leu959 and Glu957, and uses the nitrile end to approach the glycine loop (Gly882, Gly884, Gly887). Pyrrolopyrimidine or analogs thereof appear as a common feature of many JAK inhibitors [25]. The later-developed PF–06700841 (Brepocitinib, PDB: 6DBN) decomposes pyrrolopyrimidine into two independent rings and replaces the nitrile with two larger F atoms [26]. The case of 3C9 (PDB: 4QT1) is a new type of C-5 substituted pyrrolopyrazine, which is expected to serve as a potent and selective JAK3 inhibitor [27]. Linking with amino acids near Glu957 and Leu959 can fix one end of the compound on JAK, which seems to be the hinge-binding element when designing various JAK inhibitors, while the extension of the molecular length at the other end is relatively flexible. Compared with Tofacitinib, some JAK inhibitors may exhibit more malleability or linearity within their frameworks, such as TG101348 (Fedratinib) [28], CYT387 (Momelotinib) [29] and LS104 (a rare non-ATP competitive JAK2 inhibitor) [30].

## 2. Results

### 2.1. Binding Affinity and Configuration of Six Phenolic Acids from S. miltiorrhiza and Artichoke

**Figure 3:**
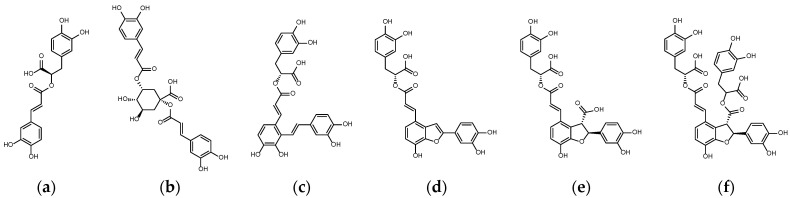
The six phenolic acids in *Salvia miltiorrhiza* and *Cynara scolymus*. (**a**) Rosmarinic acid (RosA); (**b**) Cynarin (CY); (**c**) Salvianolic acid A (SalA); (**d**) Salvianolic acid C (SalC); (**e**) Lithospermic acid (LSA); and (**f**) Salvianolic acid B (SalB).

**Figure 4:**
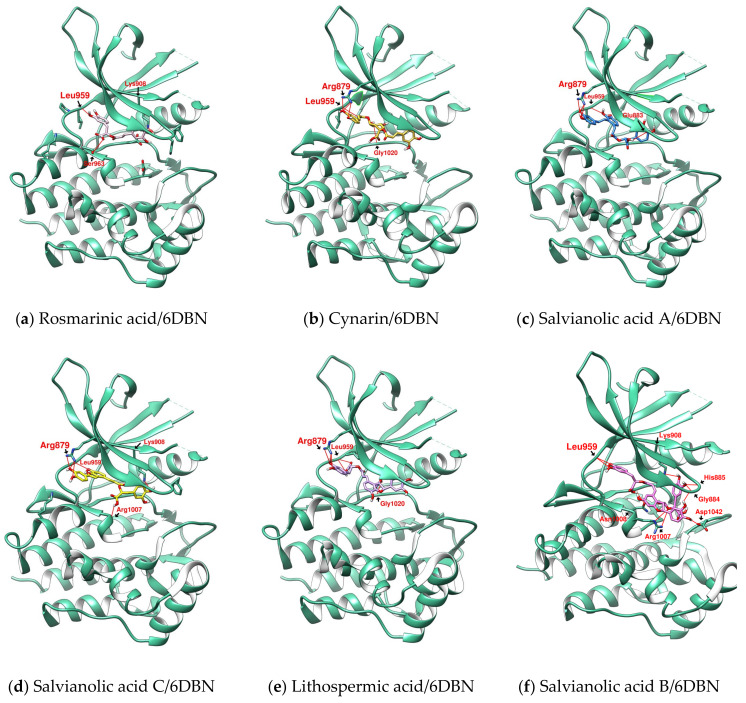

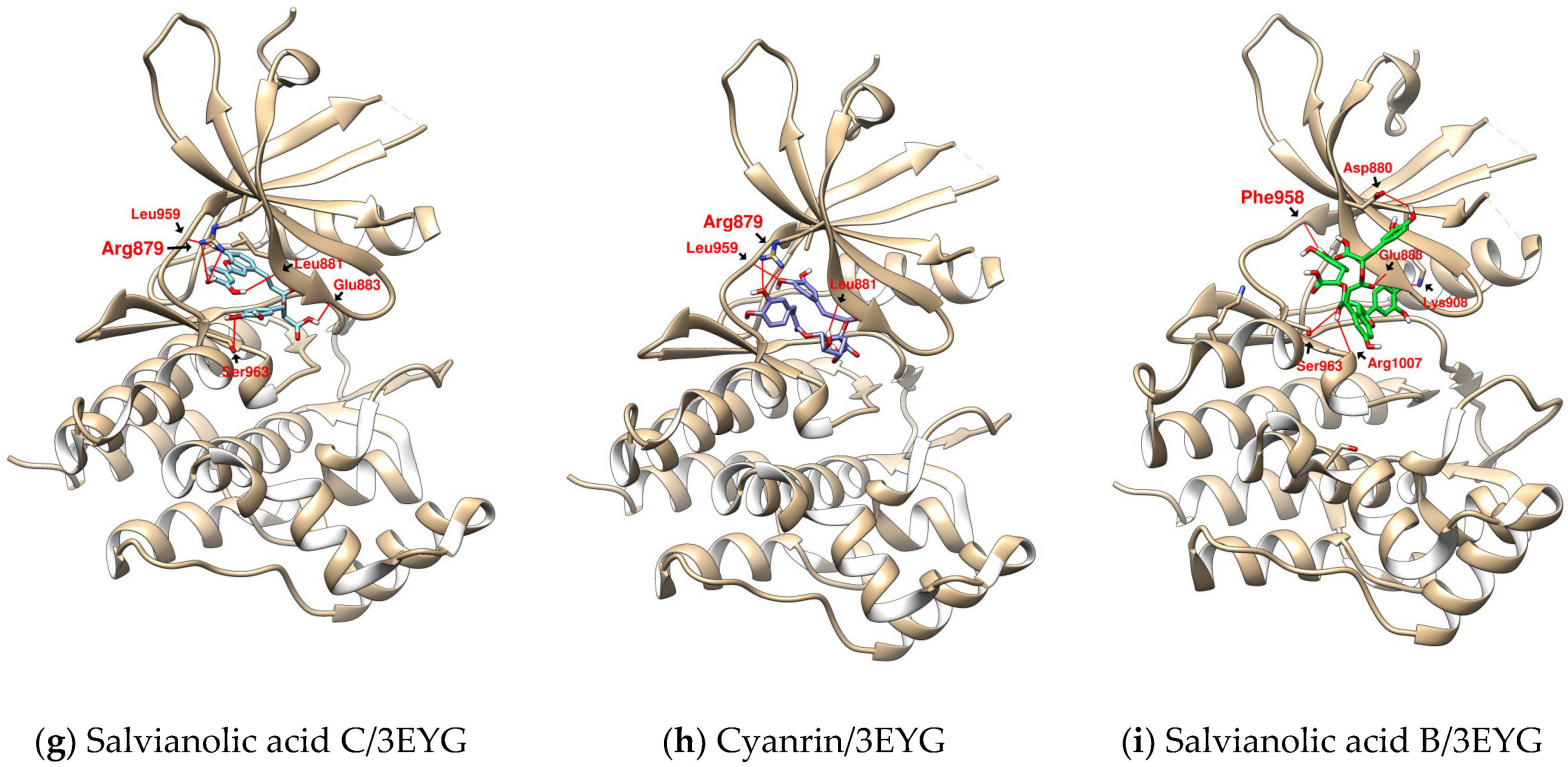
The lowest-energy configurations of six phenolic acids docked to JAKs: (**a**–**f**) docking to PDB: 6DBN, (**g**–**i**) docking to PDB: 3EYG. Hydrogen bonds were shown as red lines.

Through molecular modeling, it was found that the binding affinities of the six phenolic acids of these two plants to JAKs had the potential for JAK inhibition, with a value of −8.7 kcal/mol or better, as shown in Table 1. The six phenolic acids scored similarly for JAK1 (PDB:6DBN) and JAK3 (PDB:4QT1), with JAK3 being slightly better. However, JAK3 expression is restricted to cells of hematopoietic lineage, whereas JAK1 is widely expressed. The results for JAK1 are, thus, more relevant [31]. The two most-discussed phenolic acids in *S. miltiorrhiza*, Salvianolic acids B and A, and Cynarin in artichoke, have scores comparable to Rottlerin. Among the six phenolic acids, SalC showed the best binding affinity for JAK1, 2 and 3. Relative to PDB:6DBN, LSA and SalB were weaker in docking with PDB:3EYG. The lowest energy configuration in docking indicated that these phenolic acids sit between Leu959 and Gly884 similar to Tofacitinib or PF–06700841, and most of them can establish hydrogen bonds with Leu959 (as shown in Figure 2, Figure 3 and Figure 4). The hydrogen bond length, distance from the gatekeeper residue Met 956 [31] and hydrophobic contacts and π-interactions analyzed by PoseView are listed in Table 2.

The better-known Salvianolic acids show potential for JAK inhibition, as docking analysis reveals favorable configurations and hydrogen bonds. Salvianolic acid B is a rich phenolic acid in *S. miltiorrhiza*, but studies indicate that it may be degraded into Denshensu, RosA, LSA, SalA, C, D, E, etc., in the gastrointestinal tract and reduce oral availability [32,33]. Docking shows that some of its degradants still retained potent binding affinity to JAK, reducing concerns about the degradation of orally administered SalB. 

Compared with SalB, SalC is currently much less studied, and SalC may have more potential when discussed as an inhibitor of enzymes whose ligand is ATP. SalC has a good binding affinity for JAK. The backbone length of SalC is close to that of the JAK inhibitors TG101348 and CYT387, and all three have four rings. Cyanrin does not have the same binding affinity as SalC, but is so abundant in artichoke that it may be effective even if higher doses are required to obtain concentrations sufficient to inhibit the enzyme [23,34]. It is generally known that *S. miltiorrhiza* is the golden herb for cardiovascular, and studies have also found that it can resist thrombosis [35,36]. The relationship between the occurrence of thrombosis and the use of JAK inhibitors has attracted attention. Further research has not found a direct relationship, but it is still recommended that high-risk groups be regularly tested for related indicators [37,38]. With more research, in addition to their well-known cardiovascular and liver health benefits, these two plants may have the opportunity to be nutritional supplements for people with immune diseases or inflammation.

### 2.2. Further Analysis of the Characteristics of the Docking Configuration and Conformation

Due to the presence of ester bonds, the conformation of RosA, SalA-C, LSA and CY have a certain degree of flexibility. The structure of phenolic acids may change or degrade under the influence of pH and temperature, and structure-dependent self-association may occur in an aqueous solution [32,33,39]. Docking analysis suggests that these phenolic acids may have more than one possible configuration on the JAK (Figure 4). In AutoDock Vina, the grid setting parameters are flexible and can be adjusted according to the operator’s experience. The center coordinates of the original ligands, Tofacitinib for PDB: 3EYG and PF–06700841 for PDB: 6DBN, were set as the center of the docking grid in this study. The structural length of PF–06700841 is slightly longer than that of Tofacitinib. The docking results show that, referring to the centroid of PF–06700841, the configuration tends to extend linearly, while, referring to Tofacitinib, the skeletons may be more compact and present a U-shape. The PDB database shows that U-shape and linear shape are the common configurations of JAK inhibitors at receptors. The linear and U-shaped configurations of SalC have the same binding affinity, and the benzofuran sits similarly in both configurations. The difference is that the linear tail is attached to Lys 908 and the U-shaped tail is attached to Ser 963. The performance of the two compounds seems to be evenly matched. For larger multiaxial macromolecules such as SalB, the docking score may be weakened when part of its side chain floats out of the main active site. Therefore, the configuration of SalB on PDB:6DBN corresponds to a stronger docking affinity than on PDB:3EYG. The differences in docking with PDB: 6DBN and PDB: 3EYG suggest that the flexible structure brings complications to the relationship between phenolic acids and JAKs, increasing the difficulty of analysis. However, due to the structural flexibility of phenolic acids, when they interact with the active site of JAK, whether extended or partially folded, they can establish hydrogen bonds with multiple amino acids, thereby affecting the activity of JAK. Previously, Chen, S.C., et al. observed that SalB would inhibit the IFN-γ-induced phosphorylation of JAK2 on Tyr 1007/1008. Although the partial interaction of SalB with JAK does not act directly on the active site, the docking configuration of SalB in both PDB:3EYG and 6DBN adversely affects the phosphorylation of Tyr 1007/1008.

The docking score of the six phenolic acids to JAK1(PDB: 6DBN) increases from RosA (dimer), SalA (trimer), to SalB (tetramer), consistent with their antioxidant capacity [40]. However, SalC (trimer) is an exception because it is more effective than SalB. As suggested in the case of Tofacitinib, the hydrogen bond established with Leu959 or nearby amino acids is the key to anchoring the molecule in the ATP pocket. The lowest energy configuration of SalC docking to JAK1(PDB:6DBN) indicates that its interaction with Leu959 and Arg879 not only comes from the hydroxyl group on benzene-1,2-diol (catechol), but also from benzofuran-7-ol. The successful positioning of SalC’s benzofuran-7-ol and its catechol (benzofuran-7-ol + catechol) near Leu959 seems to be the deciding factor behind SalC achieving the best performance among the six phenolic acids. SalB and LSA have a benzofuran core similar to SalC but with one more side chain. The additional side chains provide sites for hydrogen bonding with the surrounding, but may also lead to steric interference that affects the docking results. When SalB and LSA docked with PDB:6DBN, the benzofuran core was not near Leu959, but moved towards Glu883.

## 3. Discussion

### 3.1. Analysis of the Structural Characteristics of SalC as a Potential JAK Inhibitor

**Figure 5:**
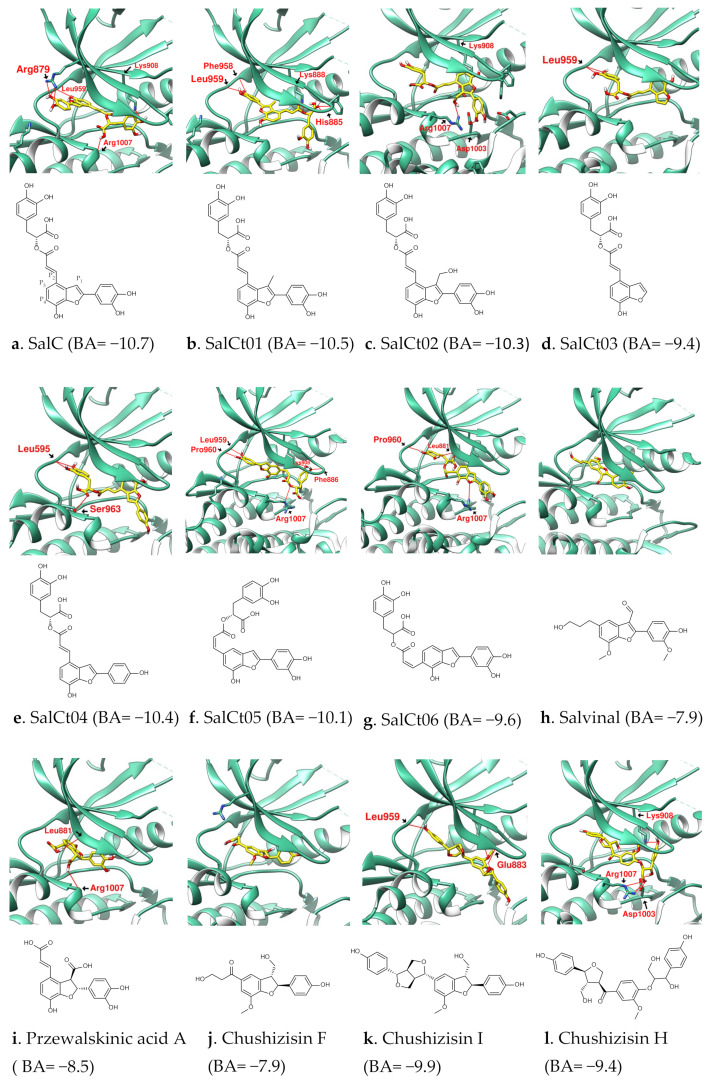
The characteristics of SalC in docking with JAK(PDB:6DBN) were found by comparing SalCt01~SalCt06. (**a**) SalC with encoding. The benzofurans of the compounds (**b**–**k**) showed different positions from SalC in the docking. (**l**) is the ring-opened form of (**k**). The location of SalC’s benzofuran brings benefits to its binding affinity. Hydrogen bonds were shown as red lines with labels.

A series of SalC derivatives and benzofuran-containing natural products were docked with JAK to compare and explore the structural features of SalC as JAK inhibitors, as shown in Figure 5. The docking results suggest that when designing benzofuran derivatives as potential JAK inhibitors, the side chain at P_1_ may affect or increase the parameters where the benzofuran is located. (1) When a short substituent was added at the P_1_ position of SalC (relative to SalC) to derive SalCt01 and SalCt02, the docking results showed that their benzofuran cores were offset from the position of SalC. Although not as good as SalC, their configuration still allows multiple interactions with JAK. (2) Salvinal and Przewalskinic acid A are rare benzofuran derivatives in *S. miltiorrhiza* with side chains at P_1_, and the position of their benzofuran core in docking is different from that of SalC [9,41]. (3) In addition to phenolic acids, many lignans also contain benzofuran cores. The antioxidant lignans Chushizisin F and Chushizisin I from *Broussonetia papyrifera* are benzofuran derivatives and have side chains at the P_1_ position [42]. In the docking results, it was observed that their benzofuran cores did not interact with Arg879. Regardless of the size of these compounds, when they have a side chain at the P_1_, it is very likely that the relationship between their benzofuran and Leu959 is different from that of SalC when docked.

There are also factors that influence where the benzofuran derivatives appear in the JAK. (4) Comparing the results of SalC and SalCt03, it was found that the catechol substituent on benzofuran also contributes to the overall positioning of SalC. For SalCt03, benzofuran lacking the catechol substituent no longer interacts with Arg879. (5) SalCt04′s case shows that if the catechol substituent is changed to phenol, the docking performance will not be as good as SalC. (6) In addition to catechol, the long side chain at P_2_ on benzofuran provides more sites for the establishment of hydrogen bonds, so SalC has a better binding affinity than Salvinal. (7) SalCt05 and SalCt06 were created by moving long side chains from P_2_ to P_3_ and P_4_ positions. Both displayed different configurations from SalC in the docking results. (8) Chushizisin H is the open-ring form of Chushizisin I. Compared with Chushizisin I, ring opening increases molecular flexibility but reduces JAK inhibition. Similarly, the binding affinity of SalC was better than that of SalA without the benzofuran ring. (9) The structural length of Chushizisin I is slightly shorter than that of SalC, but its configuration extends farther than SalC since the ester-bonded side chain of SalC on P_2_ is more flexible than the bis-tetrahydrofuran of Chushizisin I. Based on the docking results, it was observed that SalC is a potential JAK inhibitor due to its “benzofuran-7-ol + catechol”, a flexible side chain on P_2_ and no side chain on P_1_. While retaining its “benzofuran-7-ol + catechol” structure, the remaining P_2_ side chain is available for modification. The ester bond on the P_2_ side chain of SalC provides a cleavable site that can be used to attach other fragments to derive a series of new compounds. 

### 3.2. Design of SalC, RosA, CY and SalA Derivatives with Higher Binding Affinity

Natural benzofuran compounds or their derivatives have shown high medicinal value in many research fields such as cancer, inflammation, nervous system-related diseases and cardiovascular diseases. Therefore, finding natural benzofuran scaffolds for specific targets and synthesizing new benzofuran derivatives for in vitro and in vivo screening has always been an amazing project in drug design [43,44]. In molecular docking with JAK, SalC has particularly shown its research potential. As a JAK lead compound or a drug suitable for oral administration, it is expected to have high binding affinity, GI (Gastrointestinal) absorption and bioavailability. The GI absorption and bioavailability evaluated by SwissADME for SalC are low and its NH/OH ≥ 5 (≠Lipinski’s rule). However, some enzyme inhibitors may introduce more hydrogen bond donors and receptors to increase binding affinity, not necessarily following Lipinski’s rule of five. A large part of drug discovery is actually beyond Lipinski’s rule of five [45]. In drug design, there may be a choice between Lipinski’s rule, GI absorption and binding affinity.

**Figure 6:**
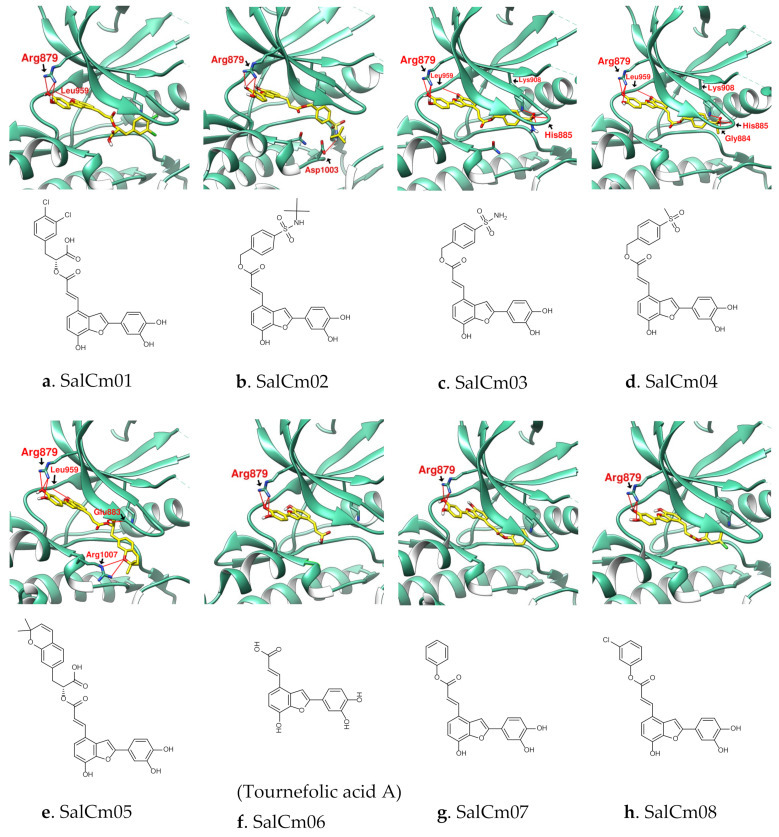
(**a**–**h**) SalCm01~SalCm08 were designed with reference to the structure of SalC in order to obtain better ADME parameters or binding affinity to JAK than SalC. Hydrogen bonds were shown as red lines with amino acid residue labels.

Further observations are given in Figure 6 and Table 3. (1) Compared with the nitrile of Tofacitinib, PF–06700841 takes double F atoms to form a bulky end group. In SalCm01′s case, one catechol of SalC was changed to orthodichlorobenzene. It was observed that the large tail end may increase the binding affinity to JAK and the chlorination also improves the bioavailability. (2) The Sulfonamide compound is a type considered in drug design. Structures derivatized with Sulfonamide have been observed in 3C9 and TG101348. SalCm02~04 are modifications of SalC with methylsulfonyl benzene or sulfonamides. Salm03 and Salm04 exhibit high JAK affinity and bioavailability, and are compounds with great research potential. (3) SalCm05 is a bicyclic structure formed by changing one end of the catechol of SalC to 2,2-dimethyl-2H-chromene. SalCm05, therefore, shows an attractive binding affinity to JAK with the value of −12.2 kcal/mol. (4) In the case of SalCm06 (Tournefolic acid A), it is shown that shortening the long side chain of SalC can improve GI absorption, but the relative binding affinity becomes weaker [46]. The molecule of Tournefolic acid A is smaller than that of Przewalskinic acid A (Figure 5), but because its benzofuran location is similar to that of SalC, it shows relatively good binding affinity. (5) SalCm07 and SalCm08 were obtained by adding phenyl group or chlorobenzene on SalCm06, which can increase the binding affinity to a value close to SalC and maintain high GI absorption. From the docking configuration of the SalCm01~08, it can be seen that the benzofuran-7-ol position of these compounds is located near Leu959, showing the stability and reproducibility of the SalC-related scaffold as a potential JAK inhibitor. SalCm01 and SalCm03~05 have very good binding affinity and bioavailability but low GI absorption. SalCm07 and SalCm08 have good binding affinity and GI absorption, but the binding affinities are not as prominent as SalCm01 and SalCm03~05. Finally, which compounds are more suitable as potential JAK inhibitors require more experiments.

**Figure 7:**
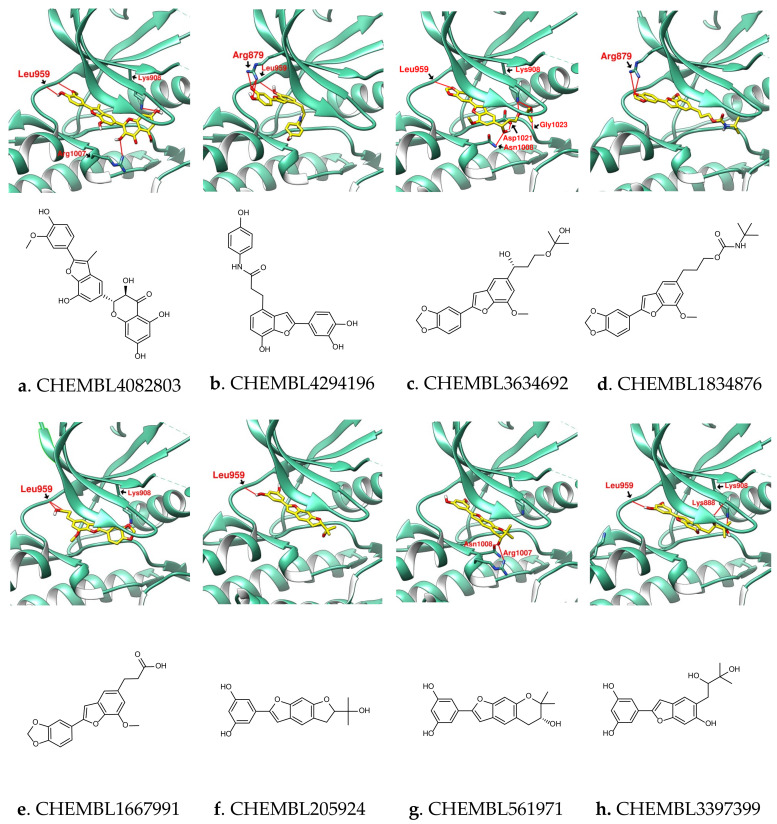
(**a**–**h**) Benzofuran derivatives with biological activity and JAK inhibition potential obtained by searching for “SalC” with SwissSimilarity. Hydrogen bonds were shown as red lines with amino acid residue labels.

Ligand-based virtual screening, such as that provided by SwissSimilarity, further provides references for drug design. By screening bioactive molecules similar to “SalC” with SwissSimilarity, a series of benzofuran derivatives were found in the ChEMB database [47]. These compounds were then exported to SwissADME and docked with JAK (PDB: 6DBN), and the compounds with drug-like properties and JAK affinity potential are recorded in Figure 7 and Table 4. The docking configuration showed that, except for the benzofuran core of CEMBL4294196, which was located at a similar position to that of SalC, the benzofuran of other molecules appeared at a position similar to that of SalCt01 (Figure 5). CHEMBL4082803 (Anhydrosilychristin) is a flavanol derivative with a benzofuran core. The interaction of its “3-hydroxy-2,3-dihydro-2-phenylchromen-4-one” and benzofuran core with the active site of JAK resulted in good docking affinity. CHEMBL205924 (Moracin O), CHEMBL561971 (Moracin P) and CHEMBL3397399 (Moracin R) are natural products isolated from *Morus alba*. These molecules, which are partially similar to SalC, again demonstrate the potential of benzofuran derivatives as JAK inhibitors. The search for phenolic acids or flavanonol derivatives with benzofuran cores may be the way to discover more compounds with potential for JAK inhibition.

**Figure 8:**
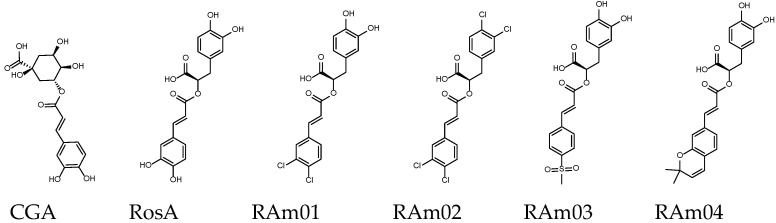
Structures of RosA derivatives. Chlorogenic acid (CCA) is for reference and comparison.

**Figure 9:**
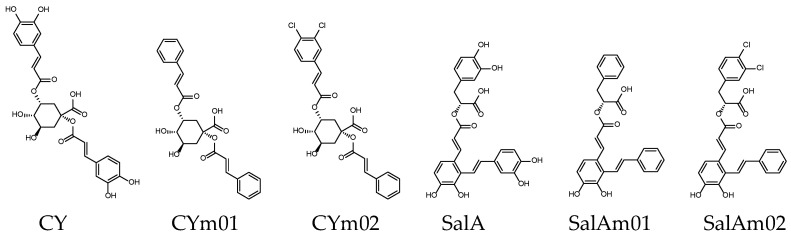
Chemical structures of Cynarin and SalA derivatives.

Rosmarinic acid (RosA) is present in many plants, SalA is the second most discussed Salvianolic acid and Cynarin is rich in artichokes. These three compounds are the subject of many medical studies. RosA, CY and SalA may also be the potential backbone of JAK inhibitors, as shown in Figure 8 and Figure 9 and Table 5 and Table 6. Similar to the method applied to SalC derivatives, the orthodichlorobenzene, methylsulfonyl benzene or 2,2-dimethyl-2H-chromene modification of RosA’s catechol has the opportunity to improve the related GI absorption, bioavailability or binding affinity. The bioavailability evaluation of RAm02 can reach 0.85, and RAm04 shows good binding affinity. In the design of CY and SalA derivatives, CYm02 and SalAm02 enhanced GI absorption while maintaining binding affinity by the hydrogenation and chlorination of catechol.

## 4. Materials and Methods

Molecular docking or molecular modeling is a computer-based drug design program that can simulate the possibility and status of ligand docking to the active site of the receptor. In this study, AutoDock Vina, designed by Dr. Oleg Trott of the Molecular Graphics Laboratory at the Scripps Research Institute, was used as a molecular modeling tool due to its fast processing and high reproducibility [50].

JAK1, JAK2 and JAK3 are included as docking receptors. The related crystal structure of JAK1/JAK2 (PDB: 3EYG), JAK1(PDB:6DBN) and JAK3(PDB:4QT1) was retrieved from the Protein Data Bank (https://www.rcsb.org/, accessed on 10 January 2020) [25,26,27]. The 2D and 3D structures of phenolic acids from *Salvia miltiorrhiza* and *Cynara scolymus*, and the reference JAK inhibitor molecules used in the text were obtained from the ZINC database and PubChem. In Figure 6, Figure 8 and Figure 9, a series of designed phenolic acid derivatives were drawn with MarvinSketch (ChemAxon) and checked for structural correctness. ChemDraw, MarvinSketch and ChemDoodle 3D (iChemLabs) are used to edit 2D and 3D structures to the required format (mol^2^) of the software before performing docking.

UCSF Chimera (http://www.rbvi.ucsf.edu/chimera/, accessed on 6 October 2019) is a program that provides an integrated platform for “Dock prep”, AutoDock Vina operation and visualization of docking results. Under UCSF Chimera 1.13.1, the protein (receptor) was prepared by removing extra water molecules followed by adding lost hydrogen atoms. “Dock prep” was applied to add charges on the receptor and ligand. Charges were computed with ANTECHAMBER [51,52]. Molecular docking and virtual screening were performed using AutoDock Vina _1_1_2 (http://vina.scripps.edu/download.html, accessed on 9 May 2020) through UCSF Chimera [50]. In the docking process, the protein was set as rigid, and the phenolic acids were considered flexible with default torsional degrees of freedom. A grid box size of 35 × 35 × 35 was defined on the structure of JAKs. The grid center coordinates of JAK1/2 (PDB:3EYG) were set to X = 9.875, Y = 12.903, Z = −13.748. The grid center coordinates of JAK1 (PDB:6DBN) were set to X = 10.945, Y= 15.005, Z = −14.57. The grid center coordinates of JAK3 (PDB:4QT1) were set to X = 3.066, Y= −15.203, Z = −5.286. After importing the prepared ligands, the molecular docking program was run with default settings. Ten sets of data within the maximum energy difference = 3 (kcal/mol) were obtained, and the lowest energy configuration with RMSD = 0 was selected as the result for analysis. Chimera’s built-in FindHBond program was used to search for hydrogen bonds between ligands and receptors, and the Relax H-bond constraints were set to 0.4 angstroms and 20.0 degrees. Additionally, PoseView (https://proteins.plus/, accessed on 28 June 2021) was used to analyze π-interactions and Hydrophobic contacts. Hydrogen bonds (shown as red lines) and markers can be displayed simultaneously with the 3D rendering of the lowest energy configuration. This is helpful for readers to quickly capture the relationship between the ligand and JAK under the stereogram. This also helps to compare the configuration and hydrogen bonding of these phenolic acids with Tofacitinib and PF−06700841 on JAK.

Through ADME (absorption, distribution, metabolism and excretion) analysis, it is possible to initially assess whether the compound has a background that can be developed into a drug. The ADME parameters of compounds were calculated by SwissADME (http://www.swissadme.ch/index.php, accessed on 18 October 2020) from the Swiss Institute of Bioinformatics (SIB) [53]. SwissSimilarity provides an interface for screening analogs of specific compounds to obtain more samples for comparison [47]. In this study, under SwissSimilarity, the FP2 method was used to screen the ChEMBL Database in bioactive groups. The series of compounds screened by SwissSimilarity will be returned to SwissADME to review their pharmacokinetic properties, druglike nature and medicinal chemistry friendliness. Compounds with good ADME parameters were then sent to AutoDock Vina for docking with JAK (PDB: 6DBN). Finally, configurational analysis and hydrogen bond labeling of compounds with good binding affinity for JAK were presented. From this process, it was found that the combined application of SIB’s SwissADME, SwissSimilarity and AutoDock Vina is a feasible method to discover JAK inhibitors with great research potential.

## 5. Conclusions

JAK is an important drug target for the treatment of immune abnormalities, but there is not much discussion of natural products focusing on JAK inhibition. For individuals with immune disorders that do not yet necessitate prescription medication, nutritional supplements may be used to improve the quality of life. *S. miltiorrhiza* and artichoke have the potential to fill this role. This molecular modeling study shows that Salvianolic acids A, B and C, Lithospermic acid and Cynarin have good binding affinity for JAK (−9.6~−10.7 kcal/mol), highlighting their suitability for further research on JAK inhibitors. Among the six phenolic acids studied, SalC had the best binding affinity. Comparing the differences between SalC and other phenolic acid backbones, it is found that the benzofuran core and side chain distribution of SalC are largely responsible for its binding affinity. Synthetic JAK inhibitors take pyrrolopyrimidine or analogs as the pharmacodynamic subunit. A benzofuran core of SalC, when docked with JAK, produces a pyrrolopyrimidine-like hydrogen-bonding interaction with Leu959 in the hinge region. Benzofurans may become a new pharmacophore in the search for natural sources of JAK inhibitors. Maintaining the characteristics of SalC with a modification of catechol to 2,2-dimethyl-2H-chromene at the other end, SalCm05 achieves a binding affinity of −12.2 kcal/mol. Hydrogenation or chlorination of RosA, CY and SalA terminal catechols can improve their GI absorption or bioavailability. When various derivatives of these structurally flexible phenolic acids are docked with JAK, there is an opportunity to find more potential JAK inhibitors through this process. Further research is merited.

## Figures and Tables

**Table 1:** JAKs’ binding affinity of six phenolic acids (unit: -kcal/mol).

	JAK1/JAK2 (PDB:3EYG)	JAK1 (PDB:6DBN)	JAK3 (PDB:4QT1)
Rosmarinic acid	−8.7	−8.8	−9.0
Cynarin	−9.6	−9.7	−9.8
Salvianolic acid A	−9.7	−9.8	−9.9
Salvianolic acid C	−10.7	−10.7	−11.0
Lithospermic acid	−9.4	−10.0	−10.1
Salvianolic acid B	−9.6	−10.3	−10.2

**Table 2:** Receptor–ligand interaction of six phenolic acids docking with JAKs.

Ligand	Docking Receptor	Closest Distance to Met 956	Hydrogen Bond the Hinge Region (Length: Å)	Hydrogen Bond N- and C-Terminal Lobes (Length: Å)	Hydrophobic Contacts	π-Interactions (π-Cation and π-π Stacking)
RosA	6DBN	4.324	L959 (3.11)	K908 (3.912)	V889	K908
				S963 (3.759)	L1010	(π-cation)
CY	6DBN	2.952	L959 (3.829)	R879 (2.955)	L881	K908
				G1020 (2.662)	G962	(π-cation)
SalA	6DBN	3.716	L959 (3.391)	R879 (2.979)	L881	-
				E883 (2.009)	G962	
SalC	6DBN	3.77	L959 (2.782)	R879 (3.074)	L881	K908
				R1007 (2.060)	G884	(π-cation)
				K908 (3.300)	G962	
					L1010	
LSA	6DBN	4.179	L959 (3.074)	R879 (2.995)	L881	K908
				G1020 (2.182)	G962	(π-cation)
SalB	6DBN	4.187	L959 (2.341)	G884 (2.328)	V889	F886 (π-π)
				H885 (2.080)	L1010	
				K908 (3.379)		
				R1007 (3.621)		
				N1008 (3.077)		
				D1042 (1.966)		
SalC	3EYG	3.849	L959 (3.050)	R879 (3.943)	L1010	-
				L881 (2.068)	A906	
				E883 (2.359)	L881	
				S963 (3.672)	V889	
					G962	
CY	3EYG	2.828	L959 (2.967)	R879 (3.052)	L1010	-
				L881 (2.704)	V889	
					G962	
SalB	3EYG	4.237	F958 (2.490)	K908 (3.158)	V889	-
				S963 (3.799)	L1010	
				R1007 (2.512)	G882	
				D880 (3.056)		
				E883 (3.086)		

1. Hydrogen bonds and bond lengths between ligands and receptors were analyzed using UCSF Chimera; π-interactions and hydrophobic contacts were analyzed using PoseView. 2. The gatekeeper residue Met 956 on the ATP binding pocket limits the acceptable ligand volume [31]. Due to the flexible structure, the configuration of these six phenolic acids in docking can be very close to Met956, and the distances are recorded in the table (unit: Å). 3. According to Williams, NK et al., the protein tyrosine kinases (PTKs) domains of JAK1/2 include (1) an N-terminal lobe (residues 865–955) with 5 β-sheets and 1 α-helix; and (2) C-terminal lobe (residues 960–1154) with eight α-helices. Between the two lobes is the substrate (ATP)-binding cleft. The hinge region (Met956, Glu957, Phe958, Leu959) is bounded between the N-terminal and C-terminal lobes [25]. The most important interaction between Tofacitinib and JAK is the two hydrogen bonds of Leu959 and Glu957. Hydrogen bonds to the hinge region are of relative importance and are therefore listed separately.

**Table 3:** SalC Derivative (SalCm01–08 series) with ADME parameters.

Name	SalC	SalCm01	SalCm02	SalCm03	SalCm04	SalCm05	SalCm06	SalCm07	SalCm08
Binding score	−10.7	−11.1	−10.7	−11.3	−11.6	−12.2	−9.4	−10.5	−10.6
MW (g/mol)	492.43	529.32	537.58	481.47	480.49	542.53	312.27	388.37	422.81
GI absorption	Low	Low	Low	Low	Low	Low	High	High	High
Lipinski’s rule violation	④	①	①	-	-	①	-	-	-
Bioavailability score	0.11	0.56	0.55	0.55	0.55	0.56	0.56	0.55	0.55

SalCm01~SalCm08 were docked with PDB 6DBN, and their ADME parameters were predicted by SwissADME. The calculation of GI absorption is based on the research of Daina A et al. [48]. Lipinski’s rule is ① MW ≤ 500, ② MLOGP ≤ 4.15, ③ N or O ≤ 10, ④ NH or OH ≤ 5. The reference value of Bioavailability score recommended by Martin YC. is “*falls from 85% if the polar surface area (PSA) is ≤ 75 Å^2^, to 56% if 75 < PSA < 150 Å^2^, to 11% if PSA is ≥150 Å^2^”* [49].

**Table 4:** ADME parameters and binding scores (to JAK) for compounds (**a**)~(**h**) in Figure 7.

Name	CHEMB-L4082803	CHEMB- L4294196	CHEMB- L3634692	CHEMB- L1834876	CHEMB-L1667991	CHEMB- L205924	CHEMB- L561971	CHEMB-L3397399
Binding score	−11.2	−9.9	−9.4	−9.3	−9.0	−9.4	−9.4	−9.4
MW (g/mol)	464.42	405.40	400.42	425.47	340.33	326.34	326.34	344.36
GI absorption	Low	High	High	High	High	High	High	High
Lipinski’s rule violation	-	-	-	-	-	-	-	-
Bioavailability score	0.55	0.55	0.55	0.55	0.56	0.55	0.55	0.55

The above compounds were docked with PDB 6DBN, and their ADME parameters were predicted by SwissADME. The calculation of GI absorption is based on the research of Daina A et al. [48]. Lipinski’s rule is ① MW ≤ 500, ② MLOGP ≤ 4.15, ③ N or O ≤ 10, ④ NH or OH ≤ 5. The reference value of Bioavailability score recommended by Martin YC. is “*falls from 85% if the polar surface area (PSA) is ≤ 75 Å^2^, to 56% if 75 < PSA < 150 Å^2^, to 11% if PSA is ≥150 Å^2^*” [49].

**Table 5:** RosA Derivative with the prediction of ADMEADME parameters.

Name	CGA	RosA	RAm01	RAm02	RAm03	RAm04
Binding score ^1^	−8.8	−8.8	−8.9	−8.9	−9.3	−10.0
MW (g/mol)	354.31	360.31	397.21	434.10	443.30	410.42
GI absorption [48]	Low	Low	High	High	High	High
Lipinski’s rule violation ^2^	④	-	-	②	-	-
Bioavailability score [49]	0.11	0.56	0.56	0.85	0.56	0.56

^1^ Binding to PDB: 6DBN (kcal/mol); ^2^ Lipinski’s rule: ① MW ≤ 500, ② MLOGP ≤ 4.15, ③ N or O ≤ 10, ④ NH or OH ≤ 5.

**Table 6:** Cynarin and SalA Derivative with the prediction of ADME parameters.

Name	CY	CYm01	CYm02	SalA	SalAm01	SalAm02
Binding score ^1^	−9.7	−9.2	−9.7	−9.8	−9.3	−9.7
MW (g/mol)	516.45	452.45	521.34	494.45	430.45	499.34
GI absorption [48]	Low	High	High	Low	High	High
Lipinski’s rule violation ^2^	①③④	-	①	④	-	-
Bioavailability score [49]	0.11	0.56	0.56	0.11	0.56	0.56

^1^ Binding to PDB: 6DBN (kcal/mol); ^2^ Lipinski’s rule: ① MW ≤ 500, ② MLOGP ≤ 4.15, ③ N or O ≤ 10, ④ NH or OH ≤ 5.

## Data Availability

Data are contained within the article.
